# Self‐reactive B cells are increased in all major stages of peripheral development in Sjögren's disease

**DOI:** 10.1111/imcb.70005

**Published:** 2025-02-17

**Authors:** Adrian YS Lee, Zhankun Qi, Katherine JL Jackson, Joanne H Reed

**Affiliations:** ^1^ Centre for Immunology and Allergy Research, Westmead Institute for Medical Research and Faculty of Medicine and Health University of Sydney Westmead NSW Australia; ^2^ Department of Immunology Westmead Hospital and Institute of Clinical Pathology and Medical Research, NSW Health Pathology Westmead NSW Australia; ^3^ Garvan Institute for Medical Research Darlinghurst NSW Australia

**Keywords:** autoantibodies, autoimmunity, B cells, self‐reactivity, Sjögren's disease, transitional B cells

## Abstract

Sjögren's disease (SjD) is a chronic autoimmune disorder characterized by increased circulating self‐reactive B cells. While many of these self‐reactive B cells emerge from the bone marrow, it is not known whether they are excluded from or enriched in specific developmental stages in the periphery. The aim of this study was to determine the immunophenotype of circulating self‐reactive B cells in SjD to inform more precise therapeutic targeting. Five major B cell populations: transitional, mature naïve, switched memory, double negative and plasmablasts were single‐cell sorted and cultured to produce IgG. Self‐reactive IgG was identified by ELISA, flow cytometry of permeabilized HEK293 cells and HEp‐2 indirect immunofluorescence. Immunoglobulin heavy chains were sequenced by Sanger and next‐generation sequencing. Compared with healthy donor controls (HCs), SjD patients had higher frequencies of naïve and CD21^low^ atypical memory B cell subsets, while antigen‐experienced B cells expressed more Ki67 and CD86. B cells recognizing intracellular self‐antigens were identified in all stages of peripheral B cell development for SjD and HCs, but frequencies of autoreactive B cells were up to 10‐fold higher in SjD. Self‐reactive transitional B cells expressed higher surface CD38 and lower surface IgM. An increase in self‐reactive B cells throughout peripheral development in SjD compared with HCs suggests that counterselection of autoantibody‐bearing B cells during central and peripheral tolerance checkpoints are reduced in SjD. Therapeutic strategies focused on depleting B cells based on B cell receptor specificity rather than the developmental stage would be more efficient to target self‐reactive B cells in SjD.

## Graphical abstract

In this article, we identify self‐reactive peripheral blood B cells in Sjögren's disease through a single‐cell culture system. We find 2‐ to 10‐fold increased frequencies of self‐reactive B cells during all stages of peripheral development in Sjögren's compared with healthy donors. This suggests a failure in both central and peripheral tolerance mechanisms contributing to self‐reactivity.

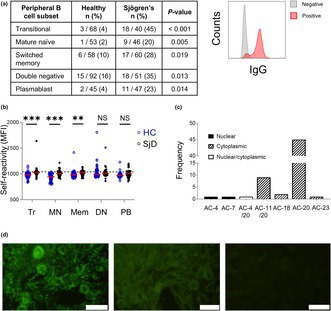

## INTRODUCTION

An archetypal chronic autoimmune disorder, Sjögren's disease (SjD), has the hallmark features of sicca symptoms, pain and fatigue. B cell hyperactivity and dysregulation play a key role in disease pathogenesis, causing hypergammaglobulinemia and the production of autoantibodies targeting the Ro/La ribonucleoprotein proteins (Ro52, Ro60, La) and rheumatoid factors (RhF).^1^ B cell depletion therapy is effective for the treatment of some severe autoantibody‐mediated manifestations of SjD (e.g. cryoglobulinemia^2^). Currently, anti‐CD20 monoclonal antibody, which targets most peripheral B cell populations with the exception of CD20^low/−^ plasmablasts, is the most common B cell depleting agent used clinically. However, the identification and immunophenotyping of self‐reactive B cells in patients may inform more precise therapy to specifically deplete self‐reactive B cells.

Advances in single‐cell and immunoglobulin sequencing have enabled the identification and study of autoantibodies and self‐reactive B cells in diverse human B cell repertoires.^3^, ^4^ A seminal paper by Wardemann *et al*.^5^ demonstrated 55–75% of bone marrow‐derived IgM^low/−^ pre‐B cells were self‐reactive to nuclear and cytoplasmic antigens in healthy human donors. In the periphery, 5–20% of mature naïve B cells were self‐reactive, which was increased to 36–67% in SjD.^6^ It is not clear whether this high proportion of self‐reactive B cells in SjD is maintained, throughout B cell development, to the antibody secreting plasmablast compartment. Therefore, we investigated single‐cell cultures of five distinct peripheral blood B cell phenotypes to identify and characterize self‐reactive clones from patients with SjD.

## RESULTS

### Naïve and CD21^low^ peripheral B cell populations are expanded in Sjögren's disease

**Figure 1:**
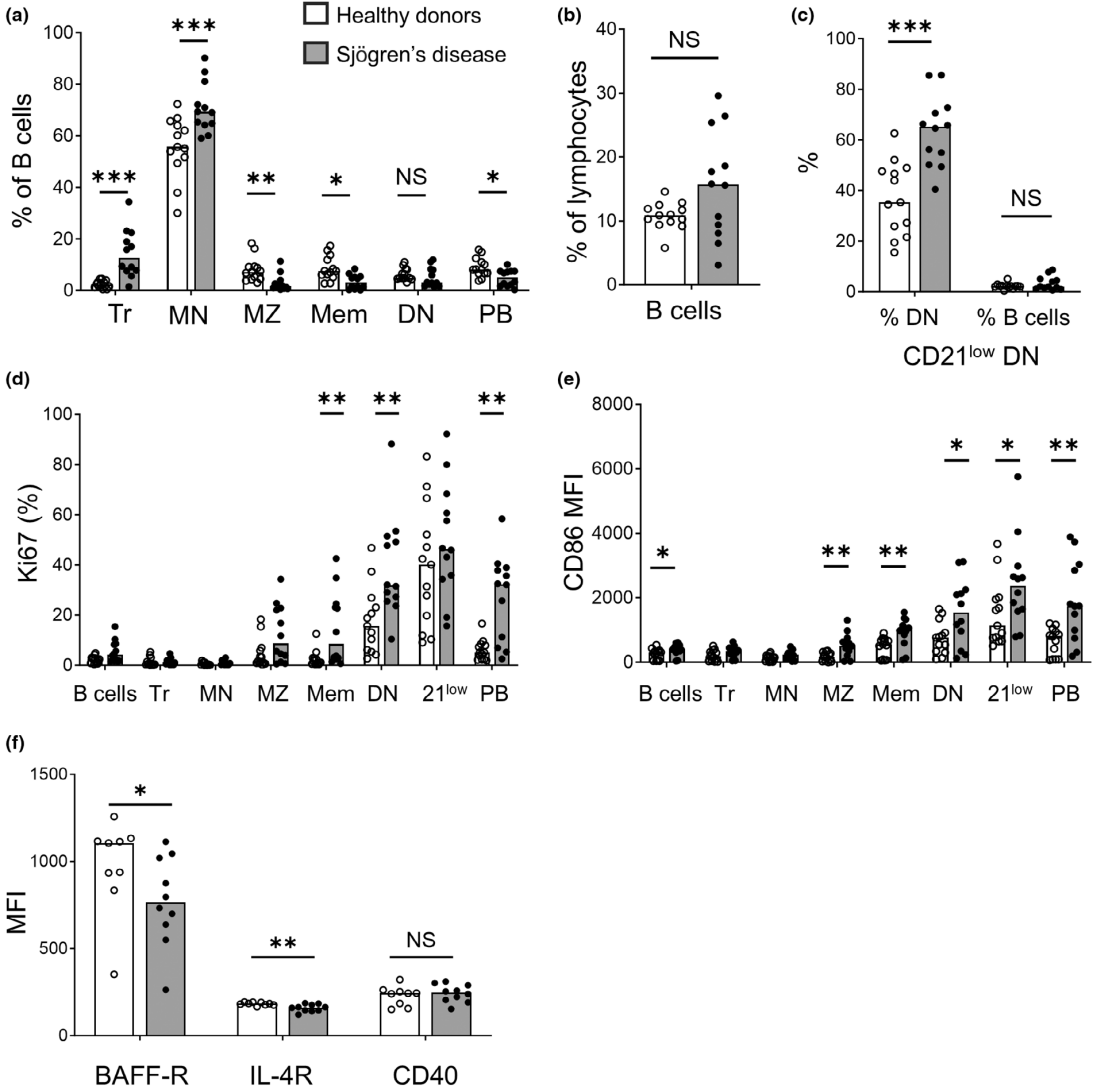
Peripheral B cell populations are altered in patients with Sjögren's disease (SjD). **(a)** Flow cytometric analyses of peripheral blood B cell populations in SjD (*n* = 12, gray bars and black circles) and healthy controls (*n* = 13, white bars and white circles). Median frequencies of live CD19^+^ B cell subsets expressed as a percentage of live CD19^+^ B cells. Transitional (IgD^+^CD27^−^CD10^+^CD38^+^), mature naïve (IgD^+^CD27^−^CD10^−^), switched memory (IgD^−^CD27^+^IgM^−^CD38^−^), double negative (DN) (IgD^−^CD27^−^) and switched plasmablasts (IgD^−^CD27^+^IgM^−^CD38^hi^). **(b)** Median frequency of live CD19^+^ events as a percentage of gated lymphocytes. **(c)** CD21^low^ DN subset frequencies expressed as either a percentage of total DN cells or CD19^+^ B cells. **(d)** Frequency of intracellular Ki67^+^ cells within each peripheral B cell subset by flow cytometry. **(e)** CD86 mean fluorescence intensity (MFI) on total peripheral B cells and subsets. **(f)** B cell activating factor (BAFF) receptor (BAFF‐R), interleukin 4‐receptor (IL‐4R) and CD40 expression on transitional B cells by flow cytometry. Figure 1 results are pooled from five independent experiments. Bars represent median values for donor group. Groups without summary statistics bar are not statistically significant from each other or labeled *NS*, not statistically significant (*P* > 0.05). *Tr*, transitional; *MN*, mature naïve; *MZ*, marginal‐zone like; *Mem*, switched memory; *DN*, double‐negative; *21* ^*low*^, CD21^low^ DN B cells; *PB*, plasmablasts. *P < 0.05, **P < 0.01, ***P < 0.001 by the Mann–Whitney *U*‐test.

Flow cytometry analysis of peripheral B cells from 12 SjD patients and 13 HCs (Supplementary figure 1a, Supplementary table 1) demonstrated increased frequencies of transitional and mature naïve B cells in SjD with decreased CD27^+^IgD^+^ marginal zone (MZ)‐like B cells, switched memory and switched plasmablasts relative to HCs (Figure 1a). There was no significant difference in total B cells (as a percentage of lymphocytes) in patients and HCs (Figure 1b). Absolute counts of B cells were unavailable since lymphocyte quantitations were not performed on all study participants, particularly HCs. The CD27^−^IgD^−^ “double negative” (DN) B cell compartment is enriched with a population expressing low levels of CD21 (Supplementary figure 1b), which have received attention for being expanded in patients with autoimmune disorders.^7^ CD21^low^ DN B cells were increased in SjD compared with HCs (Figure 1c).

### Antigen‐experienced B cells express increased Ki67 and CD86 in Sjögren's disease

We next evaluated B cell activation markers *Ki67*, an intracellular marker of cell proliferation and CD86, a co‐stimulatory molecule. Antigen‐experienced B cell subsets, DN and switched memory B cells and plasmablasts contained significantly more Ki67‐positive cells in SjD than HCs (Figure 1d). Correspondingly, all antigen‐experienced peripheral B cell subsets in SjD expressed higher levels of co‐stimulatory molecule CD86, than their HC counterparts consistent with a hyperactivated phenotype (Figure 1e).

Given that the naïve antigen‐inexperienced population is expanded in SjD (Figure 1a), we next evaluated whether SjD transitional B cells are more susceptible to the survival factors, B cell activating factor (BAFF), interleukin (IL)‐4 and CD40, by measuring receptor expression. CD40 expression was comparable, while SjD transitional B cells expressed lower BAFF‐R and IL‐4R than HC transitional B cells (Figure 1f), perhaps reflecting down‐regulation from chronic stimulation.^8^

### Self‐reactive B cells are increased in major peripheral developmental stages in Sjögren's disease

**Figure 2:**
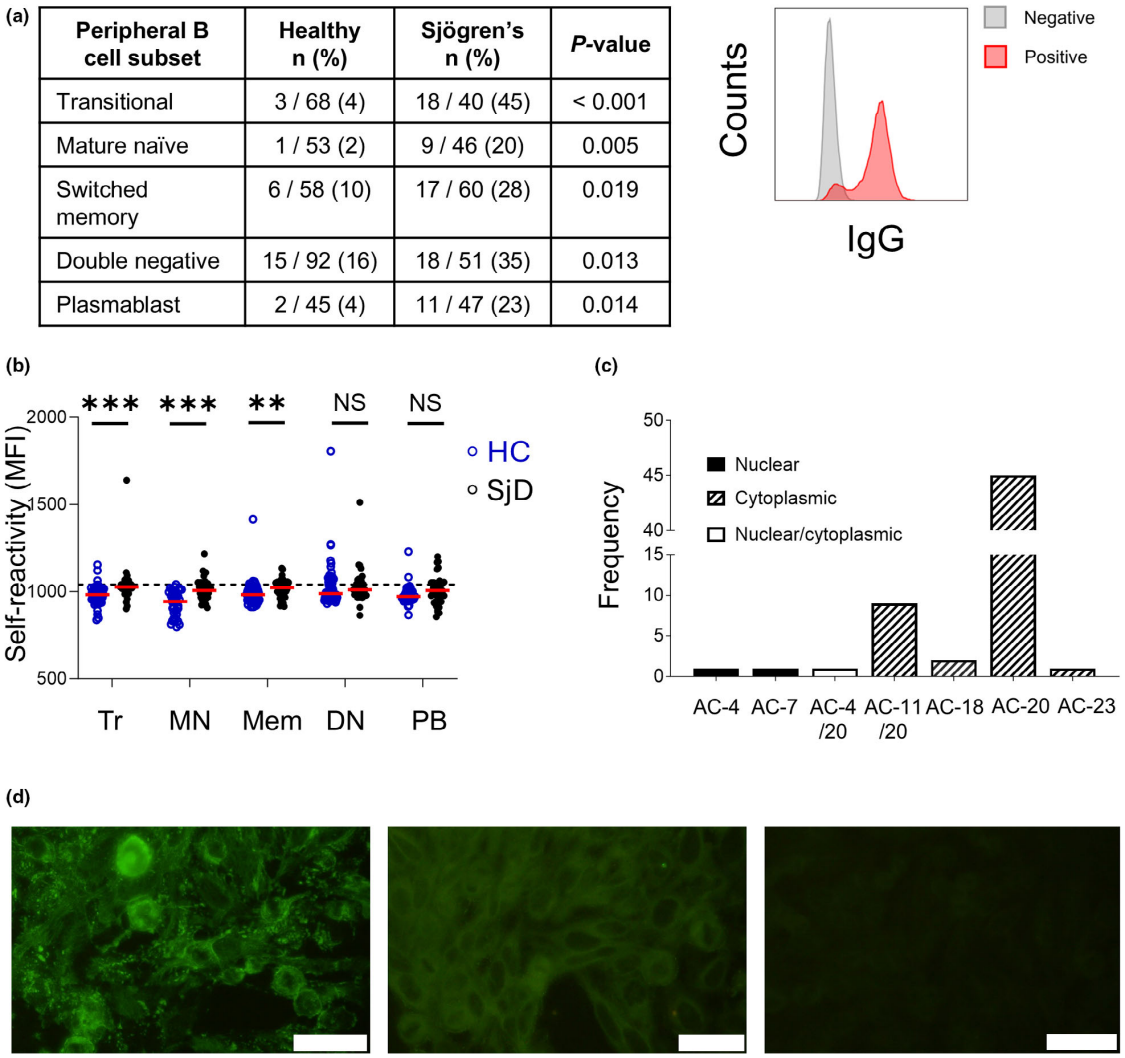
Self‐reactive B cells are prominent in every peripheral B cell subset in Sjögren's disease (SjD) and target cytoplasmic antigens. **(a)** The number of self‐reactive B cells as a proportion of IgG‐producing B cells within each peripheral subset as determined by IgG reactivity with permeabilized HEK cells by flow cytometry. Data represent pooled data from five healthy donors (HCs) and five SjD patients. Differences in proportions between each donor group were analyzed using Fisher's exact test. On the right, representative flow cytometric plot of a positive self‐reactive supernatant (red) compared with a negative supernatant (gray) is shown. **(b)** Plots of self‐reactive MFIs for permeabilized HEK self‐reactive assay; each dot represents supernatant from a single B cell clone. Median values are shown with the red bars. The horizontal dotted line represents the cut‐off value obtained from running 25 negative samples, obtaining the mean and adding 2 standard deviations. Differences were assessed using the Mann–Whitney *U*‐test. **P < 0.01. ***P < 0.001. *NS*, not statistically significant (*P* > 0.05). *Tr*, transitional. *MN*, mature naïve. *Mem*, switched memory. *DN*, double negative. *PB*, plasmablasts. **(c)** Number of antibodies producing nuclear and cytoplasmic patterns on HEp‐2 substrate immunofluorescence. Dark/black bars represent nuclear patterns, striped bars represent cytoplasmic patterns, and white bars are mixed nuclear/cytoplasmic. International anti‐cell (AC) codes for the classification of antinuclear antibody (ANA) patterns are shown. AC‐4, speckled, cell cycle‐dependent; AC‐7, few nuclear dots; AC‐4/20, fine speckled/fine cytoplasm; AC11/20, nuclear envelope/fine cytoplasm; AC‐18, glycine/tryptophan bodies; AC‐20, fine cytoplasm; AC‐23, rods and rings. **(d)** Representative micrographs of positive HEp‐2 staining from self‐reactive B cell culture supernatants (left and middle panels) and negative (non‐self‐reactive) B cell supernatant (right panel). Micrographs were taken at 40× objective lens and white bars indicate 50 μm.

Having established phenotypic differences in SjD peripheral blood B cell subsets to HCs, we next examined self‐reactivity to intracellular antigens, which is a key pathological feature of SjD.^1^ Five major peripheral B cell populations (transitional, mature naïve, memory, DN and plasmablasts) (Supplementary figure 1a) were single cell‐sorted from five SjD patients (Supplementary table 2) and five HCs and cultured for 2 weeks. The number of IgG producing B cells was equivalent in SjD (244 ± 7) and HC (316 ± 18) (*P*‐value = 0.31). However, SjD B cells at all stages of peripheral development produced significantly more self‐reactive IgG than HCs, as determined by binding to permeabilized HEK293 cells (Figure 2a, Supplementary figure 2a). Additionally, comparison of the median MFIs between each B cell subpopulation showed higher MFIs (binding to intracellular antigens) for the IgG derived from transitional, mature naïve and switched memory B cell compartments for SjD compared with HCs (Figure 2b). However, MFIs were relatively homogenous across all B cell subsets suggesting most autoantibodies detected are low‐affinity and potentially polyreactive.

Given that patients with SjD have hypergammaglobulinemia, it is possible that SjD B cells produce higher titers of IgG that contribute to higher MFIs in intracellular HEK293 self‐reactivity assays. However, this was not the case as IgG titers from cultured B cells were equivalent when comparing SjD and HC or self‐reactive and non‐self‐reactive B cell supernatants (Supplementary figure 2b, c). Finally, the MFI of IgG binding to intracellular HEK293 by flow cytometry was independent of the corresponding IgG optical density on ELISA (Spearman's ρ = 0.05, *P* = 0.796) indicating that self‐reactivity was independent of IgG titer.

To further assess self‐reactive IgG derived from peripheral B cells, the patterns of intracellular antigenic targets were evaluated by indirect immunofluorescence. Sixty of the 100 supernatants that were self‐reactive, based on flow cytometry, produced a detectable pattern on commercial HEp‐2 substrate, of which, 57 (95%) targeted cytoplasmic antigens (Figure 2c, d). Next, we tested each positive IgG supernatant derived from SjD patients for reactivity towards Ro52, Ro60 or La by ELISA. There was no reactivity towards these specific antigens in any of the SjD derived IgG supernatants (data not shown).

### Self‐reactive transitional B cells have reduced surface IgM and increased CD38 expression

**Figure 3:**
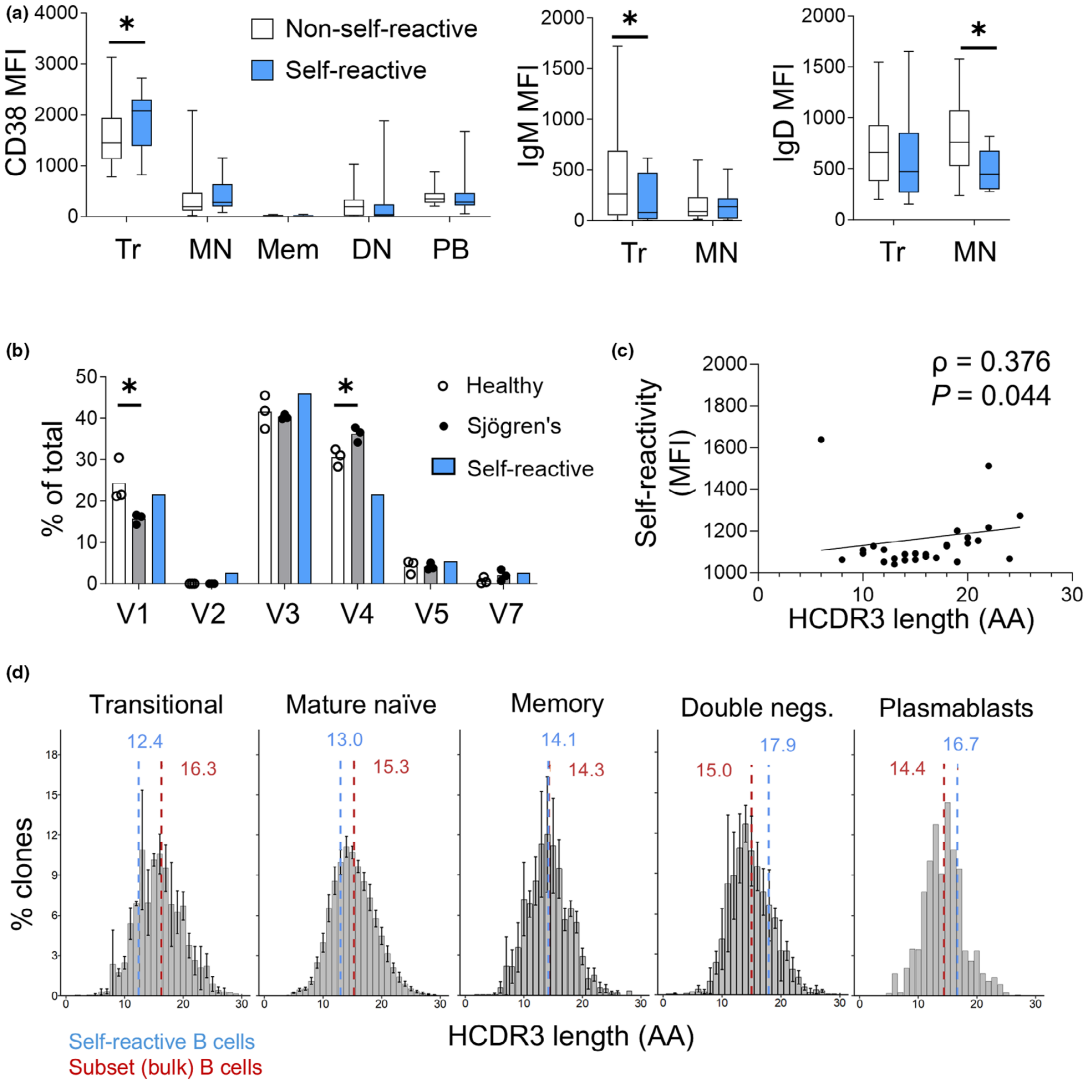
Self‐reactive transitional B cells have a distinct immunophenotype. **(a)** Expression of surface markers CD38 (left panel), IgM (middle panel) and IgD (right panel) on self‐reactive (blue) and non‐self‐reactive (white) peripheral B cell subsets by index FACS. Boxplot whiskers represent the 5th and 95th percentiles of individual values. Bar graphs without statistical asterisk summaries are not significant (*P* > 0.05). Graphs represent pooled data from eight individual experiments. *Tr*, transitional; *MN*, mature naïve; *Mem*, switched memory; *DN*, double negative; *PB*, plasmablasts. **(b)** Immunoglobulin heavy chain variable region (*IGHV*) family usage in 37 self‐reactive B cells (blue bars) compared with *IGH* repertoire sequencing of thousands of peripheral B cells from three healthy donors (white dots, white bars) and three Sjögren's disease patients (black dots, gray bars). Data for each donor were derived from the average *IGHV* subfamily usages for five B cell populations (transitional, naïve, switched memory, double negative and plasmablasts – total B cells). A minimum of 300 000 sorted cells and 250 000 sequencing reads were obtained for each B cell population per donor. V1, *IGHV1*; V2, *IGHV2*; V3, *IGHV3*; V4, *IGHV4*; V5, *IGHV5*; V7, *IGHV7*. **(c)** Correlation of heavy chain complementarity‐determining region 3 (HCDR3) length of self‐reactive B cells with corresponding IgG mean fluorescence intensity (MFI) measure of binding to intracellular HEK. *MFI*, mean fluorescence intensity. **(d)** Distribution of HCDR3 lengths (in amino acids; AA) among peripheral B cell IGH repertoires from healthy controls and SjD patients with mean HCDR3 length indicated (red line). Bars show mean percentage of clones for each HCDR3 length and error bars indicate standard deviation. Mean HCDR3 length shown for 27 self‐reactive B cells (blue line). *NS*, not statistically significant (*P* > 0.05). *P < 0.05.

By retrospective analyses of indexed FACS data, self‐reactive transitional B cells had increased CD38 and reduced surface IgM expression compared with their non‐self‐reactive counterparts. In contrast, only IgD expression was significantly reduced on self‐reactive mature naïve B cells compared with non‐self‐reactive (Figure 3a). There were no differences in CD10, CD19 or CD27 expression between self‐ and non‐self‐reactive B cells (data not shown). These data suggest that self‐reactive B cells are increased in the early transitional (T1) stage of development as T1 B cells have higher surface CD38 expression than later transitional (T2 and T3) subsets.^9^

### Self‐reactive DN B cells and plasmablasts have longer HCDR3

To determine whether self‐reactive B cells exhibit biases in immunoglobulin gene usage, sequencing of immunoglobulin heavy chain variable regions (IGHV) was performed on single self‐reactive B cell cultures and compared with matched peripheral B cell subsets from three HCs and three SjD patients. Massively parallel sequencing of *IGHV* repertoires from bulk sorted peripheral B cell subsets showed similar *IGHV* family usage across peripheral B cell subsets as demonstrated previously for healthy donors with dominance of *IGHV3*, followed by *IGHV4* and *IGHV1*.^10^ A trend for the reduction of *IGHV1* usage was noted in SjD across all peripheral B cell subsets compared with HCs, which was linked to increased usage of *IGHV4* and, to a lesser extent, *IGHV3* (Supplementary figure 3a–e).

*IGHV* Sanger sequencing was performed on self‐reactive B cells from the same three HC and three SjD donors. Thirty‐four of 37 self‐reactive B cells (91.9%) (8 from HCs, 26 from SjD donors) had adequate sequencing coverage for analysis. The self‐reactive B cells represented a highly polyclonal population with no evidence for the same clone in the matching bulk‐sorted peripheral B cell repertoire (data not shown), suggesting that these self‐reactive B cells were not clonally expanded in the peripheral blood. Each B cell culture was confirmed to contain one clone with a single IGHV and HCDR3 sequence identified for each B cell. *IGHV1*, *IGHV3* and *IGHV4* were the predominant subfamilies used by self‐reactive B cells however, *IGHV4* usage was reduced relative to the broader peripheral B cell repertoire (Figure 3b).

Next, we analyzed the length of the heavy chain complementarity‐determining region 3 (HCDR3), as longer CDR3 lengths are associated with self‐reactivity.^5^ In our dataset, the HCDR3 length was weakly correlated with the degree of self‐reactivity (IgG binding to intracellular HEK293) as measured by MFI (Figure 3c). The average HCDR3 length is 14.8 amino acids (aa);^11^ however, there were no significant differences in the amino acid length of self‐reactive B cells (15.4 aa) and the combined repertoires of peripheral B cell populations from matched donors (15.3 aa). Further comparison of HCDR3 lengths within each B cell population, revealed a trend of shorter self‐reactive B cell HCDR3 length compared with antigen‐inexperienced populations and longer HCDR3s for the self‐reactive DN and plasmablast antigen‐experienced B cells (Figure 3d).

Given that patients with SjD have increased frequencies of self‐reactive antigen‐inexperienced B cells (Figure 2a), we next investigated whether germline‐encoded autoantibodies are enriched in memory and plasmablast subsets in SjD, as described for SLE.^12^ The burden of IGH somatic hypermutation (SHM) in self‐reactive memory, DN and plasmablasts were compared with the corresponding B cell subset's IGH repertoire. Overall, the frequency of SHM was similarly distributed in self‐reactive B cells compared with their corresponding B cell population from the same patient (Supplementary figure 3f).

## DISCUSSION

In this study, we identified aberrations in the proportions, phenotype, self‐reactivity and immunoglobulin variable gene usage of SjD peripheral B cells. Consistent with our findings, increased proportions of circulating naïve B cells and reduced antigen‐experienced B cells have also been observed in other SjD studies.^13^, ^14^, ^15^ Memory B cells are increased in parotid gland biopsies from SjD patients, which may reflect retention or migration of antigen‐experienced B cells to sites of inflammation^15^ and account for their reduction in the circulation. However, if tissue‐sequestered memory B cells were the only driver of aberrant B cell proportions, it would be expected that the proportion of total circulating B cells as a percentage of lymphocytes would be reduced in SjD. This was not the case, as SjD total B cell frequencies trended higher than HCs (Figure 1b), suggesting that there are other factors increasing the number of antigen‐inexperienced B cells, such as resistance to apoptosis.

The earliest bone marrow emigrants, the CD38^high^ transitional compartment, harbored the highest proportion of self‐reactive B cells of all the peripheral subsets analyzed. These findings are consistent with the idea that impaired central tolerance results in less deletion of self‐reactive B cells in the bone marrow leading to increased early B cell output in the periphery. Previously we showed that transitional B cells with low surface IgM expression accommodated more diverse (self‐reactive) IGH repertoires than their IgM high counterparts.^16^ The self‐reactive transitional B cells from patients with SjD also had lower surface IgM expression compared with HC, which may enable escape from tolerance checkpoints and accumulation in the periphery. For SjD and HC, the proportion of self‐reactive cells in the mature naïve compartment was about half of that observed in transitional B cells. This is consistent with a selection checkpoint for self‐reactivity between the development of transitional to mature naïve B cells being active in both SjD and HCs.^16^, ^17^ This suggests that peripheral tolerance has not completely failed in SjD but rather, the threshold for negatively selecting self‐reactive B cells is impaired relative to HCs.^17^

The frequency of self‐reactivity increased in the switched and DN memory compartments relative to mature naïve B cells. The switched memory compartment of healthy donors was shown previously to harbor low‐affinity self‐reactive antibodies arising from somatic hypermutation.^18^ Moreover, DN B cells are associated with autoimmunity^19^ and enriched for self‐reactivity in both HCs and patients with autoimmune disease.^20^ Notably, for HC plasmablasts self‐reactivity declined to 4% but remained at 23% for SjD plasmablasts indicating an increased potential for serum autoantibody production in SjD. Cytokines that promote SjD B cell hyperactivity^21^ and genetic factors (e.g. polymorphisms in the human leukocyte antigens (HLA) class II and IGHV allelic variations) may contribute to the increased frequency of self‐reactive peripheral B cells in SjD.^22^

There are some important limitations to our study. Notably, our culture and sequencing studies were limited by a small sample size of SjD patients and the relatively low conversion of cultured B cells to IgG producing clones (~19%) limited our evaluation of individual subjects. This highlights the need for higher throughput single‐cell culturing approaches to validate our findings. Secondly, our culture system focused on IgG‐switched antigen‐experienced B cells and in SjD, self‐reactive IgM, for example rheumatoid factors, are also important pathological features in SjD.^4^

In conclusion, our study demonstrates that self‐reactive B cells are increased at all major developmental stages of peripheral B cells in SjD suggesting global factors (e.g. genetic, environmental) drive B cell dysregulation. Targeting the factors that promote the survival of self‐reactive B cells or targeting the self‐reactive B cell receptor (e.g. chimeric autoantibody receptor T cells^2^) would be the most efficient approach to specifically deplete self‐reactive B cells in SjD.

## METHODS

### Patients

Sixteen patients with Sjögren's disease were recruited from a single center at the Clinical Immunology and Allergy clinics at Westmead Hospital, Sydney, Australia. All patients met the 2016 SjD classification criteria,^23^ and provided signed, informed consent for participation in this study. Five SjD patients, that were anti‐Ro60^+^ seropositive and not on any immunomodulatory therapies, were selected for *in vitro* single B cell cultures (Supplementary table 2). Fourteen healthy control donors (HCs) that did not have any known chronic or autoimmune diseases at the time of recruitment were obtained from the Australian Red Cross Lifeblood service. As anonymized samples, the age and sex data were not available. Venous blood was drawn into ethylenediaminetetraacetic acid tubes and peripheral blood mononuclear cells (PBMCs) isolated by Ficoll Paque Plus (#17–1440‐03, Cytiva, Uppsala, Sweden) density centrifugation. Ethics approval for this project was provided by the Western Sydney Local Health District Research Office (ETH01030).

### Flow cytometry and fluorescence‐activated cell sorting (FACS)

The PBMCs were thawed and Fc receptors blocked (#564220, BD Biosciences, San Jose, USA). The cells were stained in 0.5% bovine serum albumin/PBS and Brilliant Stain buffer (#566349, BD Biosciences) with the following antibodies: CD3 APC‐H7 (clone SK7), CD10 BUV737 (HI10a), CD10 PE‐CF594 (HI10a), CD11c PE‐CF594 (B‐ly6), CD14 APC‐H7 (MφP9), CD19 BV421 (HIB19), CD19 BV605 (SJ25C1), CD21 BUV395 (B‐ly4), CD23 BUV395 (M‐L233), CD27 APC (O323), CD27 BV711 (O323), CD38 FITC (HIT2), CD38 PE (HIT2), CD40 PE (5C3), CD86 PE (2331), CD124 BV786 (hIL4R‐M57), CD268 PE‐Cy7 (11C1), IgD BB700 (IA6‐2) and IgM BV650 (G20‐127). Dead cells were excluded from analyses/FACS using viability dye eFluor780 (#65–0865‐14, eBioscience, San Diego, USA). For intracellular staining with Ki67 FITC (20Raj1), the cells were fixed and permeabilized using the Cytofix/Cytoperm kit (#554714, BD Biosciences). The cells were analyzed on a Fortessa cytometer (BD Biosciences) or sorted on Influx (BD Biosciences). For bulk sorted populations, FACS accuracy was consistently above 95%. Flow cytometry data were analyzed using FlowJo v10.10 software (BD Life Science, San Jose, USA).

### B cell cultures

PBMCs were single‐cell sorted into five B cell populations by first gating on CD3^−^CD14^−^CD19^+^ live events: transitional B cells (IgD^+^CD27^−^CD10^+^CD38^+^), mature naïve B cells (IgD^+^CD27^−^CD10^−^), switched memory B cells (IgD^−^CD27^+^IgM^−^CD38^−^), switched plasmablasts (IgD^−^CD27^+^IgM^−^CD38^hi^) and DN B cells (IgD^−^CD27^−^) (Supplementary figure 1a). The cells were cultured for 2 weeks with HEK293 cells expressing human CD40L (kind gift from Associate Professor Grant Logan, Children's Medical Research Institute, NSW, Australia), IL‐2 (#HIL2‐RO, Roche, Mannheim, Germany) and IL‐21 (#200–21, PeproTech, Cranbury, USA) to promote IgG secretion, as described previously.^24^ In total, five HCs and five SjD donor samples (Supplementary table 2) were sorted and cultured, culturing 300 individual B cells per donor (1500/group). Across all donors, a mean ± standard deviation of 19 ± 5% of cultured B cells produced detectable IgG on ELISA.

### Enzyme‐linked immunosorbent assays (ELISAs)

To detect total IgG or IgG specific for Ro52, Ro60 and La in single B cell cultures, ELISAs were performed by coating 96‐well MaxiSorp ELISA plates (#442404, Nunc via Thermo Fisher Scientific, Waltham, USA) with 0.5 μg mL^−1^ anti‐human IgG (#2042–01, Southern Biotech, Birmingham, USA) or 0.8 μg mL^−1^ Ro52, Ro60 and La autoantigens (Arotec Diagnostics Ltd, Lower Hutt, New Zealand) as described previously.^25^ Supernatants from single B cell cultures were diluted 1:10.

### Self‐reactive IgG assessments

Intracellular self‐reactivity was assessed using HEK/Expi293 cells (#A14527, Gibco via Thermo Fisher Scientific) as described.^26^ Briefly, the cells were plated at a density of 0.5 × 10^6^/well of a 96‐well V‐bottom plate then permeabilized with Cytofix/Cytoperm kit (BD Biosciences). Supernatants positive for IgG (as assessed by ELISA above) were added to the permeabilized cells and incubated for 30 min at 4°C, washed, followed by anti‐IgG PE‐Cy7 (clone G18‐145) for 30 min and then acquired on a flow cytometer. A mean fluorescence intensity (MFI) positivity threshold for IgG reactivity was greater than the mean MFI + 2 standard deviations of 20 IgG‐negative supernatants from single B cell cultures. The intra‐assay variation was <10%. Sera from patients with high intranuclear (anti‐double stranded DNA) and cytoplasmic IgG reactivity served as controls. For immunofluorescence (IF) assessment of positive self‐reactive supernatants, the supernatants were added neat to HEp2010 slides (Euroimmun, Lübeck, Germany) and staining was performed as per the manufacturer's instructions.

### B cell receptor (VDJ) sequencing

Heavy‐ and light‐chain immunoglobulin genes were amplified from bulk‐sorted B cell populations (Supplementary figure 1a) as described previously.^4^ IgM heavy chain (*IGHM*) was amplified for transitional and naïve B cells, whilst IgG heavy chain (*IGHG*) was amplified for memory, DN and plasmablast populations. Illumina P5 and P7 adaptors were ligated, and the samples were indexed (Nextera XT Index kit v2 Set B, Illumina, San Diego, USA) before being equimolar pooled into a library that was sequenced on Illumina MiSeq using 2×300 paired‐end reads. A minimum of 300 000 sorted cells and 250 000 sequencing reads were obtained for each population per patient. RNA was extracted from self‐reactive B cell clones derived from single‐sorted B cell cultures using the RNeasy Micro kit (#74004, Qiagen, Hilden, Germany) and reverse‐transcribed into cDNA using the iScript cDNA Synthesis kit (#1708891, Bio‐Rad, Hercules, Germany). Paired IgG heavy and light‐chain genes were amplified and Sanger sequenced as described previously.^27^

### Bioinformatics analyses

The bulk‐sorted and sequenced peripheral B cell subsets were demultiplexed as part of Illumina FASTQ generation. Paired end reads were merged using FLASH^28^ and sequences were quality filtered to a minimum of q20 and converted to FASTA using FilterSeq (version 0.7.1) from Immcantation's pRESTO toolkit.^29^ Sequences were trimmed of IGHV (forward) and IGHC (reverse) primers and the IGHC were tagged using pRESTO's MaskPrimer (version 0.7.1). Samples were dereplicated with pRESTO's CollapseSeq (version 0.7.1) and input to stand‐alone IgBLAST (version 1.21.0)^30^ for alignment against the IMGT's human germline reference directory (release 202 330–1) [https://www.imgt.org/vquest/refseqh.html]. Datasets were filtered to discard truncated alignments (germline start offset >20), sequences lacking *IGHV*, *IGHJ* and/or *CDR3* calls, and non‐productive rearrangements (stop codons or frameshifts). Clonal lineages for each individual were defined using SCOper^31^ for nucleotide *CDR3* sequences with a threshold of 0.2. *IGHV* mutation for clonal lineages was summarized as the median percent nucleotide difference from germline. FASTA sequences from Sanger traces were aligned with IgBLAST v1.21.0 and filtered using the same criteria as for bulk‐sorted datasets. The IgBLAST outputs were aggregated, filtered, summarized and visualized in R (version 4.3.0) within RStudio (version 2024.4.2.764) using the following R packages; tidyverse,^32^ scoper (version 1.3.0),^31^ shazam (version 1.2.0)^33^ and ggsci (version 3.0.3) (https://CRAN.R‐project.org/package=ggsci).

### Statistical analyses

Continuous variables were compared using the non‐parametric Mann–Whitney *U*‐test and categorical data comparisons were performed using Fisher's exact test. GraphPad Prism v10 and R v4.3 were used to construct graphs and calculate statistics. An alpha value of 0.05 was set as the statistical significance threshold.

## AUTHOR CONTRIBUTIONS

**Adrian YS Lee:** Conceptualization; data curation; formal analysis; investigation; methodology; writing – original draft. **Joanne H Reed:** Conceptualization; funding acquisition; investigation; methodology; project administration; supervision; writing – original draft. **Zhankun Qi:** Data curation; investigation; writing – review and editing. **Katherine JL Jackson:** Data curation; investigation; resources; writing – review and editing.

## CONFLICT OF INTEREST

The authors declare no conflict of interest.

## Supporting information


Supplementary figure 1.
**Supplementary figure 2**.
**Supplementary figure 3**.
**Supplementary table 1**.
**Supplementary table 2**.

## Data Availability

The data that support the findings of this study are available from the corresponding authors upon reasonable request.
