# Supplementary material for: Self‐reactive B cells are increased in all major stages of peripheral development in Sjögren's disease
Source: Immunol Cell Biol. 2025 Feb 17;103(4):401–10. doi: 10.1111/imcb.70005 (PMC11964785; doi:10.1111/imcb.70005)
Supplement: Supplementary file 1 — Supplementary figure 1. Supplementary figure 2. Supplementary figure 3. Supplementary table 1. Supplementary table 2. [file IMCB-103-401-s001.pdf]

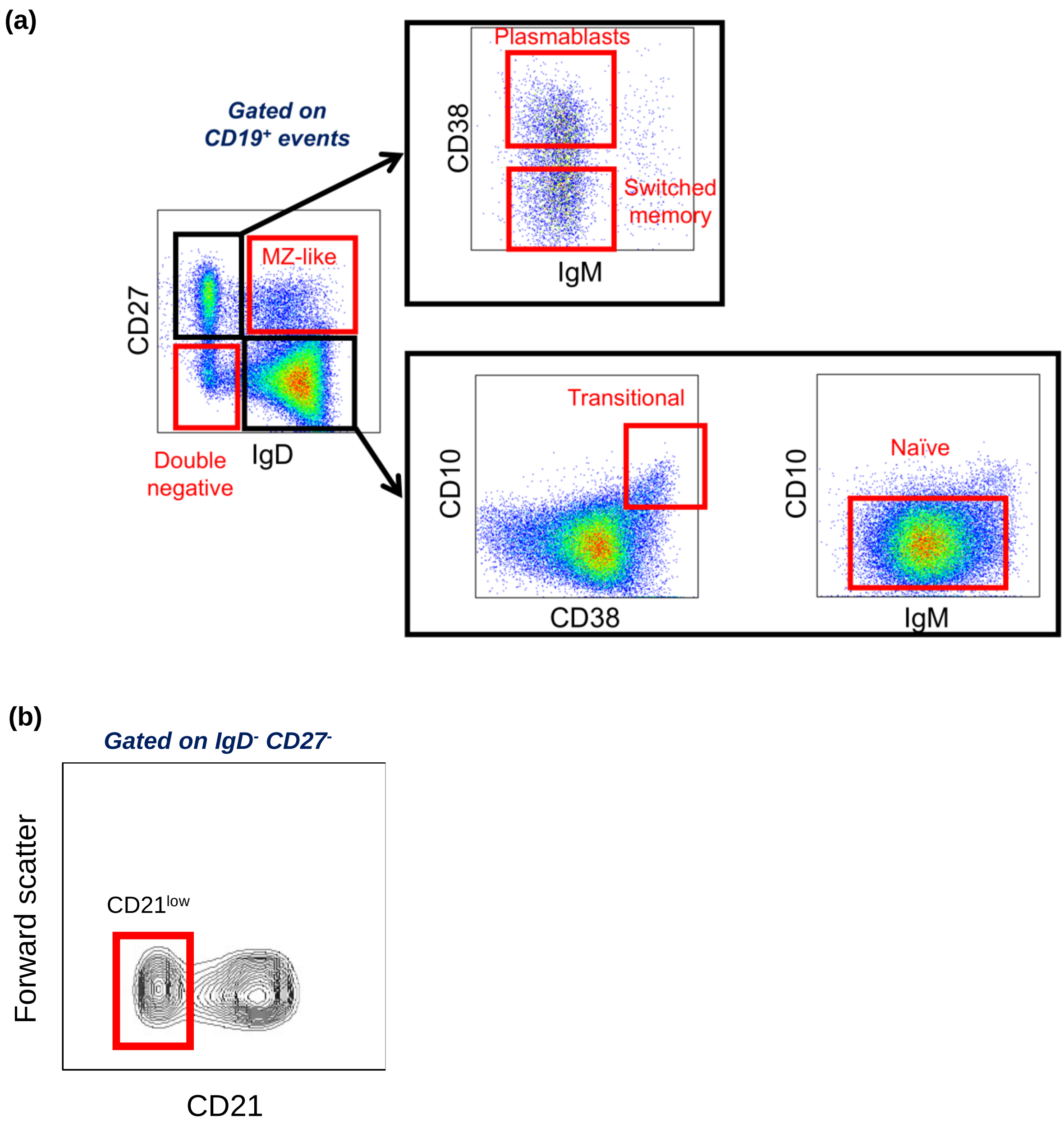

Supplementary figure 1. Gating strategy for single-cell B cell fluorescence-activated cell sorting (FACS) and flow cytometry. **(a)** The first plot on the left is gated on lymphocytes, singlets and live $CD19^{+}$ $CD3^{–}$ $CD14^{–}$. **(b)** Gating strategy for the definition of $CD21^{low}$ B cells. The plot is gated on the $IgD^{–}$ $CD27^{–}$ double negative B cell gate.

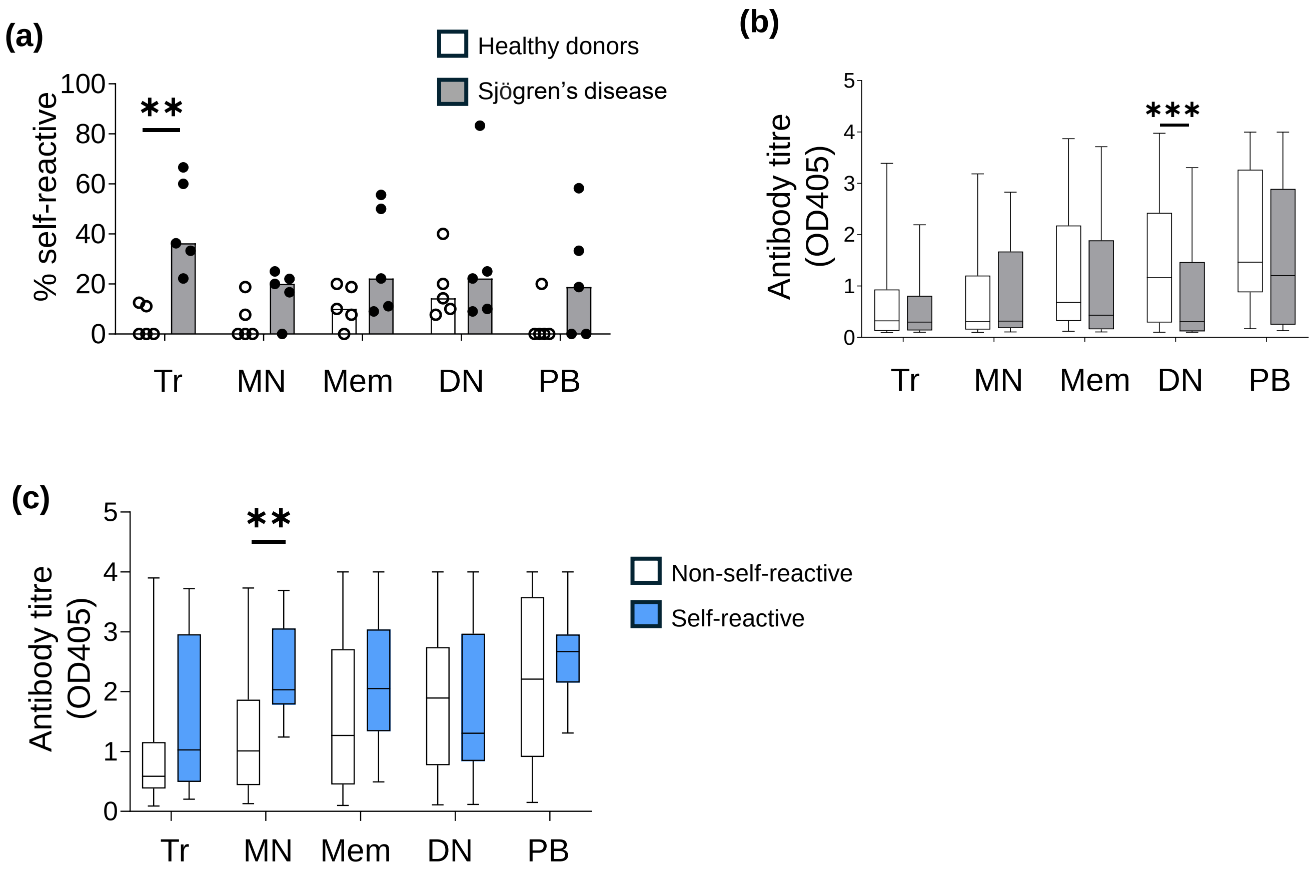

Supplementary figure 2. (a) Percentage of self-reactive B cells within each peripheral subset as determined by IgG reactivity with permeabilised HEK293 cells by flow cytometry. Columns represents median self-reactive percentages for 5 healthy donors (HCs) and 5 Sjögren's disease (SjD) patients. ** $P < 0.01$ by the Mann-Whitney *U*-test. Columns within each B cell compartment without statistical summary bars do not differ significantly from each other ($P > 0.05$). Antibody (IgG) titres measured by ELISA optical density (OD) for **(b)** all positive IgG B cell supernatants from SjD (grey bars) and HCs (white bars) and **(c)** all IgG positive supernatants from non-self-reactive (white plots) and self-reactive (striped bars) B cells. Each dot represents a single B cell culture supernatant from SjD or HCs. Box-and-whiskers without statistical summary bars are not statistically significant from each other ($P > 0.05$). ** $P < 0.01$, *** $P < 0.001$ by the Mann-Whitney *U*-test. Tr, transitional B. MN, mature naïve. Mem, memory. DN, double negative. PB, plasmablasts.

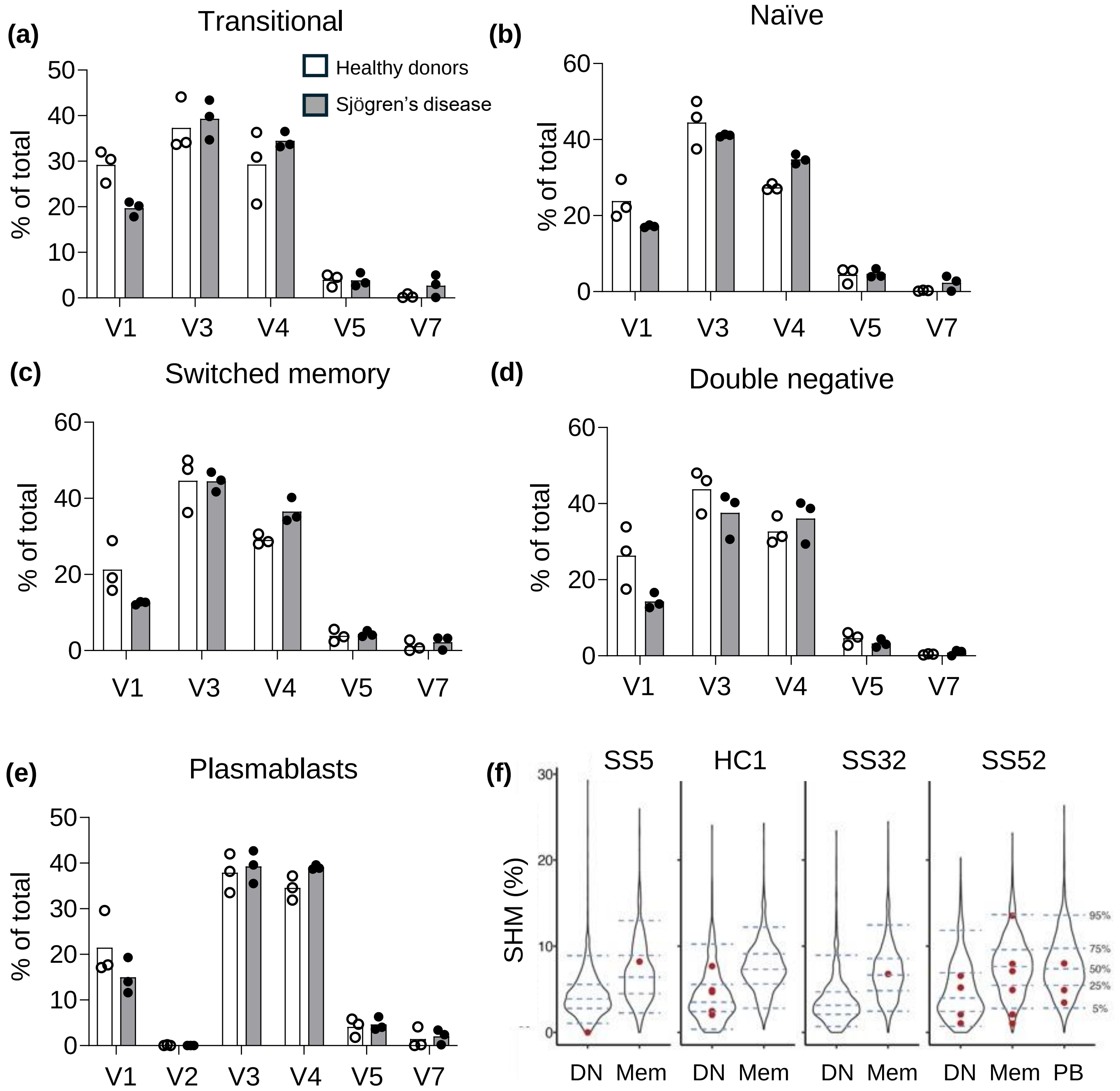

Supplementary figure 3. (a-e) *IGH* repertoire sequencing of peripheral B cell populations from patients with Sjögren's disease (SjD) (black dots, grey bars) and healthy controls (HCs) (white dots, white bars). Average percentage of total mRNA sequencing reads for each *IGHV* family member for n = 3 donors per group. Each dot represents one donor. V1, *IGHV1*. V2, *IGHV2*. V3, *IGHV3*. V4, *IGHV4*. V5, *IGHV5*. V7, *IGHV7*. **(f)** Violin plots showing the distribution of somatic hypermutations (SHM) in IGH repertoires from peripheral B cell subsets for healthy controls (HC) and Sjögren's disease (SS) overlayed with single self-reactive B cells (red dots). Blue dashed lines indicate distribution percentiles; 5, 25, 50, 75 and 95th. *DN*, double negative. *Mem*, switched memory. *PB*, plasmablasts.

Supplementary table 1. Laboratory data of the Sjögren's disease patients used in flow cytometric analyses. *ESR*, erythrocyte sedimentation rate. *ESSDAI*, EULAR Sjögren's syndrome disease activity index.

	Age (years) / Gender	IgG (g/L)	IgA (g/L)	IgM (g/L)	Rheumatoid factor (IU mL^{-1}) (< 16)	ESR (mm hr^{-1})	ESSDAI
**SS2**	70 / Male	8.8	0.5	0.5	32	-	2
**SS10**	35 / Female	7.2	3.1	3.9	238	-	2
**SS13**	36 / Female	19.9	3.4	0.9	33	20	1
**SS31**	33 / Female	14.1	2.9	0.6	15	8	0
**SS53**	63 / Male	31.5	5.5	1.8	151	73	2
**SS57**	46 / Female	12.9	4.0	1.5	11	8	0
**SS65**	28 / Female	21.2	2.8	0.8	10	12	4
**SS67**	44 / Female	18.7	3.6	0.9	96	17	1
**SS73**	47 / Female	27.5	4.1	1.7	80	33	2
**SS74**	42 / Female	18.3	2.7	0.6	10	23	1
**SS82**	42 / Female	14.2	2.3	1.5	18	7	2

Supplementary table 2. Clinical details of the five seropositive Sjögren's disease (SjD) patients included for single cell B cell cultures. *ESSDAI*, EULAR Sjögren's syndrome disease activity index.

	SS52	SS5	SS32	SS45	SS86
**Gender**	Female	Female	Female	Female	Female
**Age (years)**	53	34	50	81	26
**Serology**	Anti-Ro60	Anti-Ro52<br>Anti-Ro60<br>Anti-La	Anti-Ro60	Anti-Ro52<br>Anti-Ro60	Anti-Ro52<br>Anti-Ro60
**Rheumatoid factor**	Not detected	Detected	Not detected	Not detected	Detected
**Ocular sicca (subjective)**	Yes	Yes	Yes	Yes	Yes
**Oral sicca (subjective)**	Yes	Yes	No	Yes	Yes
**Extra-glandular manifestations**	Arthralgias<br>Fatigue	Arthralgias<br>Fatigue<br>Alopecia<br>Low C4<br>Hypergammaglobulinaemia<br>Cryoglobulinaemia	Arthralgias<br>Fatigue	(Nil)	Arthralgias<br>Fatigue<br>Hypergammaglobulinaemia
**ESSDAI**	0	4	2	0	4
